# Smartphone-Based Ecological Momentary Assessment Among Community-Dwelling Older Adults: Observational Feasibility and Acceptability Study

**DOI:** 10.2196/94949

**Published:** 2026-07-08

**Authors:** Xuan Liang, Su Hyun Park

**Affiliations:** 1 Saw Swee Hock School of Public Health National University of Singapore Singapore Singapore; 2 National University Health System Singapore Singapore

**Keywords:** ecological momentary assessment, feasibility, compliance, acceptability, older adults, mobile Health, mHealth

## Abstract

**Background:**

Ecological momentary assessment (EMA) enables repeated, real-time measurement of emotional states, behaviors, and contextual exposures in individuals’ daily lives. Although EMA has been increasingly used in health and behavioral research, evidence regarding the feasibility, compliance, and acceptability of smartphone-based EMA among older adults in Asian settings remains limited.

**Objective:**

This study aimed to evaluate the feasibility, EMA compliance, usability, and acceptability of a 14-day smartphone-based EMA protocol among community-dwelling older adults in Singapore.

**Methods:**

The data came from the Ecological Momentary Assessment in Ageing study, a smartphone-based EMA study designed to assess mental well-being, lifestyle behaviors, and perceived neighborhood environment among older adults. Adults aged 65 years or older in Singapore were enrolled and asked to complete 5 EMA prompts per day over 14 consecutive days using a smartphone app. Each EMA prompt remained available for up to 5 hours after delivery. EMA prompts assessed momentary mental well-being, sleep, physical activity, screen time, and perceived neighborhood environment. Feasibility indicators included recruitment and retention rates. EMA compliance was assessed as the proportion of completed EMA prompts out of all scheduled prompts. Usability and acceptability were assessed using a poststudy survey.

**Results:**

Between June 2025 and December 2025, a total of 186 individuals initially expressed interest in the study, of whom 139 (74.7%) scheduled an appointment, and 132 (95%) of the latter attended the appointment. Of those who attended, 98.5% (130/132) were enrolled. Participants had a mean age of 70.1 (SD 4.04) years; 65.4% (85/130) were female, 90.8% (118/130) were Chinese, and 65.4% (85/130) had postsecondary or university-level education. All enrolled participants completed the 14-day EMA protocol, resulting in 100% (130/130) retention. Across all participants, 99% (9007/9100) of the scheduled EMA prompts were completed. The poststudy usability survey was completed by 96.9% (126/130) of the participants. Among those who completed the poststudy usability survey, 88.1% (111/126) agreed or strongly agreed that the app was easy to use, 84.1% (106/126) agreed or strongly agreed that most people would learn to use it quickly, and 87.3% (110/126) preferred the app over face-to-face interviews. Overall, 91.3% (115/126) reported being satisfied or strongly satisfied with the app, and 100% (126/126) indicated willingness to be contacted for future research.

**Conclusions:**

A 14-day smartphone-based EMA protocol was highly feasible and acceptable among community-dwelling older adults in Singapore, with high retention and compliance and favorable usability ratings. These findings support the use of EMA methodologies in aging research within Asian contexts and suggest that older adults can successfully engage with repeated smartphone-based assessments when protocols are appropriately designed and supported.

## Introduction

As populations age worldwide, the increasing demographic representation of older adults and the associated burden of multimorbidity and psychological distress have become major public health concerns [1,2]. In Singapore, one of Asia’s fastest-aging societies, 23.8% of the population is projected to be aged 65 years or older by 2030 [3]. This demographic shift underscores the urgent need for targeted strategies to support mental well-being in later life. According to the Institute of Mental Health, approximately 1 in 27 older adults (aged ≥60 years) in Singapore experienced depression in 2013 [4], with an anticipated rise in these figures following the psychological impacts of the COVID-19 pandemic [5,6]. Unaddressed psychological distress in older adults is associated with an elevated risk of adverse health outcomes, including cardiovascular disease, dementia, and premature mortality [7-11]. Managing these risks is further complicated by the high prevalence of multi-morbidity in this population, which can exacerbate both psychological and physical health challenges and increase the complexity of monitoring health outcomes in daily life.

Ecological momentary assessment (EMA) has emerged as a novel measurement approach involving repeated measurements of individuals’ behaviors, emotions, or perceptions in their natural environment [12]. Unlike traditional surveys, EMA captures real-time data, allowing for ecologically valid insights into the moment-to-moment fluctuations in daily experience. Previous studies have demonstrated the potential benefits of EMA in capturing within-person variability in cognitive, emotional, and physiological measures among older adults [13,14]. EMA has also been increasingly applied among older adults with chronic conditions, including cardiovascular disease and cancer, for which real-time data collection offers particular advantages for monitoring symptom burden and daily functioning [15-18].

A review by Kim et al [19] assessed the feasibility of EMA among older adults aged 65 years and older across 38 studies. Although some studies reported dropouts or nonadherence due to technical difficulties or health-related issues, these challenges were not unique to older age, suggesting that, with careful protocol design and appropriate digital tools, EMA can be feasible and effective in older populations [19]. A recent meta-analysis further reported generally high compliance among older adults participating in EMA studies [20]. In addition to feasibility, EMA offers an important methodological advantage as it is less susceptible to recall bias and allows for the collection of repeated, real-time information on current exposures and experiences [21]. Its ability to capture environmental, situational, and psychological factors concurrently provides a more comprehensive understanding of daily life among older adults.

Although EMA and time-sampling studies have been conducted among middle-aged and older adults in Asian populations, including studies of affective, social, and psychophysiological processes [22-24], fewer studies have focused primarily on the implementation of smartphone-based EMA protocols in older adults. Existing studies differ in study aims, participant age ranges, assessment platforms, prompt schedules, and response windows, making direct comparison of feasibility and adherence difficult. In addition, engagement with smartphone-based EMA may vary according to digital literacy, technology adoption, daily routines, and comfort with mobile data collection. Therefore, implementation-focused evidence on feasibility, compliance, and acceptability remains important for informing future EMA studies involving older adults in Asian settings. This study aimed to evaluate the feasibility, EMA compliance, usability, and acceptability of a 14-day smartphone-based EMA protocol among community-dwelling older adults in Singapore, a highly urbanized city-state in Asia.

## Methods

### Study Design

This observational feasibility study was conducted as part of the Ecological Momentary Assessment in Ageing study, which was designed to examine daily mental well-being and related lifestyle and neighborhood contextual factors among older adults. Participants completed repeated real-time assessments of emotional states, lifestyle behaviors, and perceived neighborhood environment while going about their daily routines. Feasibility was assessed using recruitment and retention rates. EMA compliance was assessed using prompt- and participant-level completion metrics. Usability and acceptability were assessed using poststudy survey measures of app usability, satisfaction, preference compared with face-to-face interviews, and willingness to be contacted for future research.

### Participants and Recruitment

Participants were recruited between June 2025 and December 2025 from the National University of Singapore (NUS) community, with additional recruitment from the Singapore Population Health Studies (SPHS). Recruitment within the NUS occurred via targeted email invitations sent to faculty and staff. Eligible SPHS participants who had previously consented to be contacted for future research received email invitations through the SPHS team in compliance with the Personal Data Protection Act 2012.

Eligible participants were Singapore citizens or permanent residents aged 65 years or older who were able to read English and owned a smartphone (iOS or Android) with an active data plan. Participants were required to download and use a smartphone app during the 14-day study period. Individuals were excluded if they had a diagnosis of schizophrenia, psychotic depression, or severe dementia or screened positive for possible cognitive impairment using the Singapore version of the Abbreviated Mental Test (AMT) [25]. Age-adjusted cutoffs were applied (≤7 for the ages of 65-74 years; ≤6 for ≥75 years). The AMT items can be found in Multimedia Appendix 1.

The original study protocol aimed to recruit 150 participants, anticipating approximately 20% attrition, yielding a target analytic sample of 120 participants. A total of 130 participants were enrolled. As this was a descriptive, implementation-focused study rather than a hypothesis-testing study, this sample was considered sufficient to summarize recruitment, retention, EMA compliance, usability, and acceptability.

### Study Procedures

Before participant recruitment, the EMA protocol and RealLife Exp app were internally pretested by 2 study team members, each using the app over a 14-day testing period. Pretesting assessed app installation, prompt timing, reminders, survey flow, item clarity, response options, estimated completion time, and the data export process. Exported pilot data were reviewed to confirm variable meanings, time stamp fields, response coding, and how missing responses were recorded. Any uncertainties regarding app functions or exported data fields were clarified with the app provider before participant enrollment.

**Figure 1:**
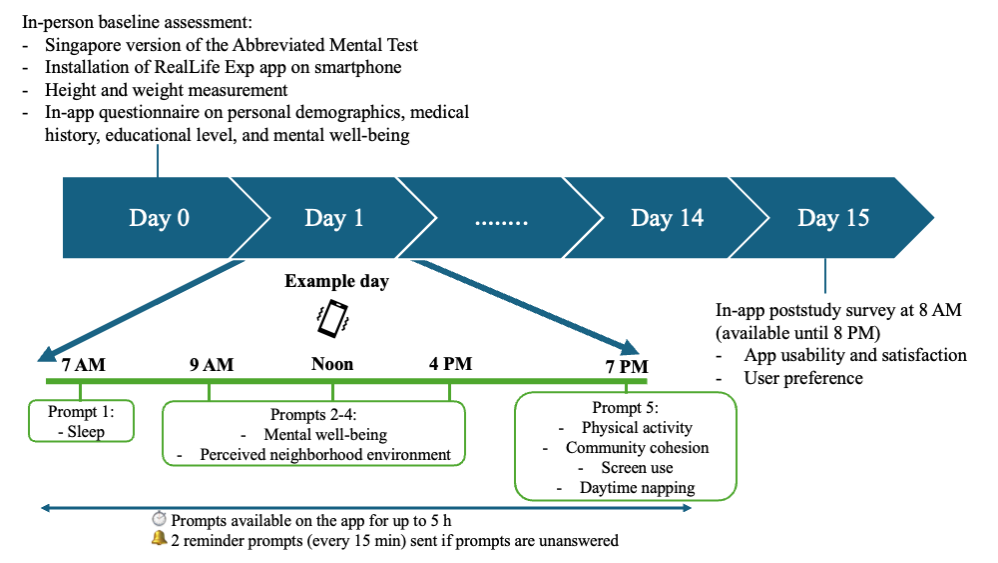
Overview of the Ecological Momentary Assessment in Ageing study protocol, including the baseline visit, 14-day ecological momentary assessment data collection period, daily prompt schedule, response window, and poststudy survey.

The detailed study protocol, including the baseline visit, 14-day EMA data collection period, daily prompt schedule, response window, and poststudy survey, is summarized in Figure 1. Enrolled participants attended an in-person baseline visit at the study center. During this visit, study staff confirmed eligibility, administered the Singapore version of the AMT, measured height and weight, and guided participants through the installation and use of the RealLife Exp app on their own smartphones. This included assistance with downloading the app, accessing the survey package using a one-time passcode provided by the research team, navigating the survey interface, enabling push notifications, and allowing the app to run in the background where required. No account registration or user profile creation was required.

Participants completed a baseline questionnaire through the app that collected information on demographic characteristics, medical history, educational level, and baseline mental well-being. Details of the baseline assessment items and response options are provided in Multimedia Appendix 2. Before leaving the study center, participants were instructed on how to access and respond to EMA prompts, navigate survey items, review incomplete responses, manually open available surveys if a push notification was missed, and confirm successful submission before exiting the app. Participants were reminded that each EMA prompt allowed for only 1 access session and that prompts should be completed and submitted before closing the app. They were also informed that the number and content of items varied by prompt type, with responses selected from predefined options such as Likert-type scales or multiple-choice categories. Participants had the opportunity to ask questions and practice using the app before beginning the EMA protocol.

Participants then completed the 14-day EMA data collection period using the RealLife Exp app. Upon completion of the study protocol, participants completed a brief poststudy survey within the app. The 6-item survey assessed app usability, preference compared with face-to-face interviews, overall satisfaction, and willingness to be contacted for future research. App usability items assessed participants’ perceived likelihood of frequent use, ease of use, and learnability of the app. Usability and preference items used 5-point agreement scales ranging from “strongly disagree” to “strongly agree.” Overall satisfaction was assessed using a 5-point scale ranging from “strongly dissatisfied” to “strongly satisfied,” and willingness to be contacted for future research was assessed using a “yes” or “no” item.

### EMA Measures and Prompt Schedule

Participants received 5 fixed-time EMA prompts per day over 14 consecutive days. Each EMA prompt remained available for up to 5 hours after delivery, after which the prompt expired. If a prompt was unanswered, up to 2 reminder notifications were sent at 15-minute intervals.

The first prompt was delivered at 7 AM and assessed sleep quality and sleep duration from the previous night. The second, third, and fourth prompts were delivered at 9 AM, noon, and 4 PM, respectively, and assessed momentary emotional states and perceived neighborhood environment. Emotional state items included tiredness, happiness, worry, stress, loneliness or isolation, boredom, and overall mental well-being at that moment. Perceived neighborhood environment items assessed perceived safety, nearby green space or park availability, loud noise or disruptions, social connection with neighbors or community members, convenience for walking or exercise, and overall neighborhood rating. The fifth prompt was delivered at 7 PM and assessed daily physical activity, community interaction, screen use, “screen eating” (eating while using or watching screen-based devices), and daytime napping. Detailed EMA items and response options are provided in Multimedia Appendix 3, and the app-based survey screens are provided in Multimedia Appendix 4.

### EMA Compliance

EMA compliance was calculated at the prompt level as the proportion of completed EMA prompts out of the total scheduled EMA prompts during the 14-day study period. Each participant was scheduled to receive 70 EMA prompts, corresponding to 5 prompts per day over 14 days. Overall compliance was calculated across all scheduled prompts among all participants. Participant-level compliance was also calculated as the number of completed prompts divided by the number of scheduled prompts for each participant. Study staff monitored response patterns daily. Per protocol, participants who missed 3 consecutive days of EMA prompts were to be contacted to address potential technical or engagement issues; however, no participants required this follow-up during the study period.

### Ethical Considerations

Ethics approval was obtained from the institutional review board of the NUS (protocol number NUS-IRB-2024-169). All participants provided written informed consent before taking part in the study. Participant information was handled in accordance with institutional privacy and confidentiality requirements. Data collected through the RealLife Exp app were exported and stored securely within an institution-approved cloud-based storage platform (NUS Dropbox). Access to the study data was restricted to authorized members of the research team and protected via institutional log-in credentials and password authentication. Study data were analyzed in deidentified form. Participants who completed the full 14-day EMA protocol, including the poststudy survey, received SGD 100 (SGD 1=US $0.77 as of June 25, 2026) via electronic transfer. Participants who completed at least 7 consecutive days but did not complete the full study received prorated reimbursement based on the number of days completed. Participants who completed fewer than 7 consecutive days were not eligible for reimbursement.

### Statistical Analysis

Descriptive statistics were used to summarize participant characteristics, recruitment and retention rates, EMA compliance, and poststudy survey measures of usability and satisfaction. EMA compliance was summarized overall and at the participant level, as described above. Means and SDs were reported for continuous variables, and frequencies and proportions were reported for categorical variables. Additional descriptive analyses were conducted to contextualize EMA compliance and response quality. Response latency was calculated as the time between EMA prompt delivery and survey initiation among completed prompts. Prompt completion time was calculated as the time between survey initiation and survey submission. We summarized median response latency; median prompt completion time; and the proportions of completed prompts submitted within 15 minutes, 1 hour, and 5 hours of prompt delivery. Extremely short prompts were defined as prompts with a completion time of less than 10 seconds. As an indicator of potential repetitive responding, straight-lining was assessed among same-scale emotional state items administered in the daytime EMA prompts. Straight-lining was defined as selecting the same response option across all emotional state items within a prompt. Noncompleted prompts were included in the denominator for compliance calculations and excluded from response timing and response quality analyses. No imputation was performed. All analyses were conducted using Stata (version 18; StataCorp). This study was reported with reference to the CHERRIES (Checklist for Reporting Results of Internet E-Surveys) guidelines [26]. The completed CHERRIES checklist is provided in Multimedia Appendix 5.

## Results

### Recruitment and Retention

Recruitment and retention outcomes are summarized in Table 1. Between June 2025 and December 2025, a total of 186 individuals initially expressed interest in the study and were contacted via phone to confirm eligibility and explain the study procedures. Of these individuals, 74.7% (139/186) scheduled an appointment at the study site, and 95% (132/139) attended the scheduled appointment. In total, 1.5% (2/132) of the individuals who attended the appointment ultimately declined participation: one due to privacy concerns related to electronic reimbursement through the app (eg, PayNow) and one due to an inability to commit to the EMA response window. A total of 98.5% (130/132) of the participants who attended the appointment were enrolled, and all completed the 14-day EMA protocol, resulting in 100% (130/130) retention (Table 1).

**Table 1:** Recruitment, retention, and ecological momentary assessment (EMA) compliance outcomes from initial expression of interest to completion of the 14-day smartphone-based EMA protocol among community-dwelling older adults in Singapore.

			Values
**Recruitment and retention, n/N (%)**			
	Scheduled appointment among those who expressed interest	139/186 (74.7)	
	Attended appointment among those scheduled	132/139 (95)	
	Enrolled among those who attended the appointment	130/132 (98.5)	
	Completed 14-d protocol among enrolled participants	130/130 (100)	
**EMA compliance**			
	Total prompts scheduled per person	70 EMA prompts per participant (5 prompts per day × 14 d)	
	Total scheduled EMA prompts, n	9100	
	Completed EMA prompts, n/N (%)	9007/9100 (99)	
	EMA prompts not completed, n/N (%)	93/9100 (1)	
	Participant-level compliance rate (%), mean (SD)	99.0 (2.23)	
	Participants with ≥80% compliance, n/N (%)	130/130 (100)	

### Participant Characteristics

Baseline characteristics of the 130 enrolled participants are summarized in Table 2. Among the 130 participants, the mean age was 70.1 (SD 4.04) years, and participants were predominantly female and of Chinese ethnicity. Most participants (85/130, 65.4%) had postsecondary or university-level education, and slightly more than half (75/130, 57.7%) used Android devices. In total, 20.8% (27/130) reported a medical history of stroke, myocardial infarction, kidney failure, or cancer. The mean AMT score was 9.8 (SD 0.42).

**Table 2:** Baseline characteristics of community-dwelling older adults enrolled in the 14-day smartphone-based ecological momentary assessment study in Singapore (N=130).

		Values
Age (y), mean (SD)		70.1 (4.04)
**Sex, n (%)**		
	Male	45 (34.6)
	Female	85 (65.4)
**Ethnicity, n (%)**		
	Chinese	118 (90.8)
	Malay	5 (3.8)
	Indian	7 (5.4)
**Educational level, n (%)**		
	Secondary or lower	45 (34.6)
	Postsecondary	51 (39.2)
	University	34 (26.2)
**Smartphone type, n (%)**		
	iOS	55 (42.3)
	Android	75 (57.7)
**Medical history of stroke, myocardial infarction, kidney failure, or cancer, n (%)**		
	Yes	27 (20.8)
	No	103 (79.2)
BMI (kg/m^2^), mean (SD)		23.85 (3.48)
AMT^a^ score (possible range 0-10), mean (SD)		9.8 (0.42)

^a^AMT: Abbreviated Mental Test.

### EMA Compliance

EMA compliance outcomes are summarized in Table 1. Across 130 participants, a total of 9100 EMA prompts were scheduled over the 14-day study period. Of these 9100 prompts, 9007 (99%) were completed. The mean participant-level compliance rate was 99.0% (SD 2.23%), and all 130 participants exceeded the 80% compliance threshold.

Additional response timing and response quality metrics are summarized in Multimedia Appendix 6. Among completed EMA prompts, the median response latency was 11.1 (IQR 1.0-33.7) minutes, and 85.4% (7696/9007) of the prompts were completed within 1 hour of delivery. Straight-lining across same-scale emotional state items was uncommon at the prompt level, occurring in 1.1% (61/5460) of the daytime EMA prompts.

### Usability and Acceptability

Poststudy survey data on usability and acceptability were available for 96.9% (126/130) of the participants; analyses were therefore restricted to these participants. Poststudy survey responses indicated high usability and acceptability of the EMA app (Table 3). Most participants agreed or strongly agreed that the app was easy to use and that most people would learn to use it quickly. A total of 87.3% (110/126) preferred the app over face-to-face interviews, and overall satisfaction was high. All participants indicated willingness to be contacted for future research.

**Table 3:** Acceptability and usability of the RealLife Exp app among participants who completed the poststudy survey (n=126).

Item		Participants, n (%)
**I think that I would like to use this product frequently.**		
	Strongly agree	4 (3.2)
	Agree	64 (50.8)
	Neutral	55 (43.7)
	Disagree	2 (1.6)
	Strongly disagree	1 (0.8)
**I thought the product was easy to use.**		
	Strongly agree	47 (37.3)
	Agree	64 (50.8)
	Neutral	15 (11.9)
	Disagree	0 (0)
	Strongly disagree	0 (0)
**I imagine that most people would learn to use this product very quickly.**		
	Strongly agree	31 (24.6)
	Agree	75 (59.5)
	Neutral	18 (14.3)
	Disagree	2 (1.6)
	Strongly disagree	0 (0)
**Compared to a face-to-face interview, I prefer to use the RealLife Exp app.**		
	Strongly agree	43 (34.1)
	Agree	67 (53.2)
	Neutral	9 (7.1)
	Disagree	6 (4.8)
	Strongly disagree	1 (0.8)
**How would you rate your overall satisfaction with the RealLife Exp app?**		
	Strongly satisfied	46 (36.5)
	Satisfied	69 (54.8)
	Neutral	11 (8.7)
	Dissatisfied	0 (0)
	Strongly dissatisfied	0 (0)
**Would you be willing to be contacted for future research studies related to the topic?**		
	Yes	126 (100)
	No	0 (0)

## Discussion

### Principal Findings

This study demonstrated that a 14-day smartphone-based EMA protocol is highly feasible and acceptable among community-dwelling older adults in Singapore. Retention and prompt-level compliance were high, and poststudy survey findings indicated favorable usability and satisfaction with the RealLife Exp app. Although high completion rates alone do not necessarily indicate a positive user experience or meaningful engagement with every prompt, the combination of high EMA compliance, favorable usability ratings, and limited evidence of repetitive responding supports the feasibility and acceptability of the protocol in this population. The finding that all poststudy survey respondents were willing to be contacted for future research further suggests that participation in the 14-day EMA protocol was not perceived as overly burdensome and that participants may remain receptive to similar app-based research.

Several protocol features may have contributed to the high compliance observed. These included structured in-person onboarding with guided app installation and hands-on training, brief prompts using predefined response options, daily monitoring by study staff, and access to technical assistance throughout the study period. The flexible 5-hour response window may also have supported completion by accommodating older adults’ daily routines. However, response timing analyses suggested that participants generally responded well before the end of the full response window. Together, these findings highlight the importance of interpreting EMA compliance in relation to protocol design and implementation procedures.

### Comparison to Prior Work

The compliance rate observed in this study was higher than rates reported in previous EMA studies involving older adults. A systematic review and meta-analysis reported a pooled compliance rate of 86.41% (95% CI 77.38%-92.20%) across EMA studies of older adults and identified common challenges including survey burden, technical difficulties, and participant fatigue [20]. A recent pooled analysis of community-dwelling older adults further found that EMA compliance varied by age, marital status, and prompt timing, with lower compliance in the evening than in the morning and afternoon [27]. In contrast, this study observed consistently high compliance across all 5 daily prompt timings, including the evening lifestyle prompt delivered at 7 PM. This may reflect the low-burden and well-supported nature of the EMA protocol. Motivational and social factors may also be relevant to engagement in EMA studies among older adults. Prior qualitative studies suggest that older adults’ research participation may be shaped by civic responsibility, perceived contribution to community well-being, and peer networks and social support [28,29]. Although these factors were not directly assessed in this study, they may be important considerations for future EMA research involving older adults.

Our favorable usability and acceptability findings are broadly consistent with prior literature demonstrating that older adults can engage successfully with smartphone-based EMA when protocols are perceived as usable and adequately supported [30-33]. Favorable user experiences and positive usability ratings have been reported across diverse older adult populations, including those with lower income [30], cognitive and emotional difficulties [32], and depression with multi-morbidity [33]. Importantly, this study extends this evidence by showing that most participants preferred smartphone-based EMA over traditional face-to-face interviews. This finding adds to the literature by highlighting participant preference for assessment modality, which has been less frequently examined in EMA feasibility studies. However, this finding should be interpreted cautiously as prior research suggests that older adults’ preferences for digital vs face-to-face approaches are mixed, with documented barriers including digital literacy concerns, privacy, and satisfaction with existing services [34,35].

### Implications

These findings have implications for the design of future EMA and mobile health studies involving older adults. Our results highlight the importance of reporting implementation details, including pretesting procedures, prompt design, response windows, participant support, response timing, and quality metrics, to improve the interpretation and comparability of EMA studies in older populations. The findings may also inform the development of future ecological momentary interventions aimed at supporting healthy aging.

### Limitations

Several limitations warrant consideration. First, the study sample consisted of English language–literate, smartphone-owning, community-dwelling older adults willing to participate in app-based research, which may limit generalizability to older adults with poorer health status, lower digital literacy, limited access to mobile technologies, or more severe functional or cognitive limitations. Second, this study was conducted in Singapore, a highly urbanized city-state in Asia; engagement with mobile health apps may differ across cultural or geographic contexts. Third, the EMA protocol was implemented over a relatively short 14-day period; longer monitoring durations may present different challenges for participant adherence and engagement. Fourth, participant reimbursement may have contributed to the high retention and compliance observed, potentially limiting generalizability to noncompensated settings. Fifth, although response timing and straight-lining metrics suggested timely response and limited evidence of repetitive responding, these are indirect indicators and may not fully capture whether each response reflected participants’ momentary experiences. Future studies may benefit from incorporating attention checks or consistency checks to more rigorously assess response quality. Finally, as with all self-report EMA designs, responses may be subject to reactivity or social desirability bias, whereby repeated assessments may influence participants’ awareness of their behaviors or experiences and potentially affect how they report them in real time.

### Conclusions

The Ecological Momentary Assessment in Ageing study demonstrates that smartphone-based EMA is feasible and acceptable among community-dwelling older adults in Singapore. High compliance, complete retention, and positive usability ratings indicate that older adults can successfully engage with repeated real-time data collection using mobile technologies. By enabling repeated real-time assessment of lifestyle behaviors, neighborhood perceptions, and mental well-being in daily life, EMA offers a valuable research approach for aging populations and may inform the future development of ecological momentary interventions aimed at promoting healthy aging.

## Appendix

Multimedia Appendix 1 Abbreviated Mental Test Singapore version.

Multimedia Appendix 2 Baseline assessment questionnaire.

Multimedia Appendix 3 Ecological momentary assessment schedule and items.

Multimedia Appendix 4 Daily ecological momentary assessment prompts (1-5) administered via the RealLife Exp app.

Multimedia Appendix 5 CHERRIES checklist.

Multimedia Appendix 6 Ecological momentary assessment (EMA) response timing and response quality metrics among completed EMA prompts.

## Abbreviations

AMT: Abbreviated Mental Test
CHERRIES: Checklist for Reporting Results of Internet E-Surveys
EMA: ecological momentary assessment
NUS: National University of Singapore
SPHS: Singapore Population Health Studies

## Data Availability

The data that support the findings of this study are not publicly available due to ethical and privacy restrictions but may be obtained from the corresponding author on reasonable request and subject to institutional approval and applicable data protection requirements.

## References

[1] Reynolds CF 3rd, Jeste DV, Sachdev PS, Blazer DG (2022). Mental health care for older adults: recent advances and new directions in clinical practice and research. World Psychiatry.

[2] Zhou J, Song D, Ma J, Zhang G, Wu C, Chen Q, Zeng L (2023). Research trends in the mental health and multimorbidity of older people from 2002 to 2022: a bibliometric analysis via CiteSpace. Front Psychiatry.

[3] (2013). A sustainable population for a dynamic Singapore: population white paper. Strategy Group Singapore.

[4] P V A, Abdin E, Roystonn K, Devi F, Wang P, Shafie S, Sagayadevan V, Jeyagurunathan A, Chua BY, Tan B, Vaingankar JA, Yao F, Magadi H, Ma S, Chow WL, McRone P, Prince M, Mahendran R, Ng LL, Chong SA, Subramaniam M (2025). Tracking the prevalence of depression among older adults in Singapore: results from the second wave of the well-being of Singapore elderly study. Depress Anxiety.

[5] Piltch-Loeb R, Merdjanoff A, Meltzer G (2021). Anticipated mental health consequences of COVID-19 in a nationally-representative sample: context, coverage, and economic consequences. Prev Med.

[6] Sarangi A, Javed S, Karki K, Kaushal A (2021). COVID-19-associated PTSD in the elderly—lessons learned for the next global pandemic. Middle East Curr Psychiatry.

[7] Barry V, Stout ME, Lynch ME, Mattis S, Tran DQ, Antun A, Ribeiro MJ, Stein SF, Kempton CL (2020). The effect of psychological distress on health outcomes: a systematic review and meta-analysis of prospective studies. J Health Psychol.

[8] Domènech-Abella J, Mundó J, Haro JM, Rubio-Valera M (2019). Anxiety, depression, loneliness and social network in the elderly: longitudinal associations from The Irish Longitudinal Study on Ageing (TILDA). J Affect Disord.

[9] Valtorta NK, Kanaan M, Gilbody S, Ronzi S, Hanratty B (2016). Loneliness and social isolation as risk factors for coronary heart disease and stroke: systematic review and meta-analysis of longitudinal observational studies. Heart.

[10] Kuiper JS, Zuidersma M, Oude Voshaar RC, Zuidema SU, van den Heuvel ER, Stolk RP, Smidt N (2015). Social relationships and risk of dementia: a systematic review and meta-analysis of longitudinal cohort studies. Ageing Res Rev.

[11] Mushtaq R, Shoib S, Shah T, Mushtaq S (2014). Relationship between loneliness, psychiatric disorders and physical health? A review on the psychological aspects of loneliness. J Clin Diagn Res.

[12] Shiffman S, Stone AA, Hufford MR (2008). Ecological momentary assessment. Annu Rev Clin Psychol.

[13] Crawford JL, English T, Braver TS (2022). Incorporating ecological momentary assessment into multimethod investigations of cognitive aging: promise and practical considerations. Psychol Aging.

[14] Maher JP, Rebar AL, Dunton GF (2018). Ecological momentary assessment is a feasible and valid methodological tool to measure older adults' physical activity and sedentary behavior. Front Psychol.

[15] Lukkahatai N, Benjasirisan C, Huang X, Wu H, Kawi J, Zhang J, Campbell CM, Johnson CM, Christo PJ, Thrul J (2026). Smartphone-based ecological momentary assessment of pain in older adults undergoing auricular point acupressure for chronic low back pain: secondary analysis of a randomized controlled trial. JMIR Form Res.

[16] Palmen B, van Herck M, Goërtz YM, Ebadi Z, Deng Q, Thong MS, Burtin C, Peters JB, Sprooten RT, Bischoff EW, Wouters EF, Vercoulen JH, Houben-Wilke S, Janssen DJ, Spruit MA, Vaes AW (2026). Real-time symptom ratings using ecological momentary assessment versus traditional questionnaires in patients with chronic obstructive pulmonary disease: observational study. JMIR Med Inform.

[17] Bui Q, Kaufman KJ, Pham V, Lenze EJ, Lee JM, Mohr DC, Fong MW, Metts CL, Tomazin SE, Wong AW (2022). Ecological momentary assessment of real-world functional behaviors in individuals with stroke: a longitudinal observational study. Arch Phys Med Rehabil.

[18] Brusseau M, Gallet-Suchet B, Dray G, Gendrault S, Harguem L, Boiché J (2025). Motivation toward physical activity and nutrition in older cancer patients: the MONAGE protocol using ecological momentary assessment and accelerometers. BMC Geriatr.

[19] Kim H, Kim S, Kong SS, Jeong YR, Kim H, Kim N (2020). Possible application of ecological momentary assessment to older adults' daily depressive mood: integrative literature review. JMIR Ment Health.

[20] Yao L, Yang Y, Wang Z, Pan X, Xu L (2023). Compliance with ecological momentary assessment programmes in the elderly: a systematic review and meta-analysis. BMJ Open.

[21] Dunton GF (2017). Ecological momentary assessment in physical activity research. Exerc Sport Sci Rev.

[22] Chu ST, Fung HH, Chu L (2020). Is positive affect related to meaning in life differently in younger and older adults? A time sampling study. J Gerontol B Psychol Sci Soc Sci.

[23] Liu H, Chen B, Wang Y, Zhao X, Hu J (2022). Social affiliation moderates the link between depressive symptoms and heart rate variability in healthy middle-aged and older individuals: an intensive ecologic momentary assessment study. Psychophysiology.

[24] Fang B, Li D, Chen B, Huang J, Hou Y, Liu H (2022). Perceived support protects against negative affective experiences of momentary solitude: an ecological momentary assessment study. J Gerontol B Psychol Sci Soc Sci.

[25] Sahadevan S, Lim PP, Tan NJ, Chan SP (2000). Diagnostic performance of two mental status tests in the older Chinese: influence of education and age on cut-off values. Int J Geriatr Psychiatry.

[26] Eysenbach G (2004). Improving the quality of web surveys: the Checklist for Reporting Results of Internet E-Surveys (CHERRIES). J Med Internet Res.

[27] Compernolle S, Vetrovsky T, Maes I, Delobelle J, Lebuf E, De Vylder F, Cnudde K, Van Cauwenberg J, Poppe L, Van Dyck D (2024). Older adults' compliance with mobile ecological momentary assessments in behavioral nutrition and physical activity research: pooled results of four intensive longitudinal studies and recommendations for future research. Int J Behav Nutr Phys Act.

[28] Boutilier B, Warner G, Wolfe B, Askari S, Moody E, Ghanouni P, Packer T (2025). Engaging community-dwelling older adults in research: qualitative substudy of factors impacting participation. JMIR Form Res.

[29] Collazo-Castiñeira P, Rodríguez-Rey R, Cruz-Jentoft AJ, Ben Allouch S, Eglseer D, Schoufour J, Topinková E, Weijs PJ, Boirie Y, Sánchez-Izquierdo M (2025). Tailoring mHealth for healthy aging: focus group study with retirement-age adults. JMIR Mhealth Uhealth.

[30] Malkowski OS, Dunton GF, Townsend NP, Kelson MJ, Western MJ (2026). Feasibility and acceptability of 7-day smartphone-based, activity-triggered ecological momentary assessment among low-income older adults. Innov Aging.

[31] Kennedy-Malone L, Hevel DJ, Sappenfield KB, Scheer H, Zecca C, Maher JP (2021). Low-income, older African Americans' engagement in and perceptions of a smartphone-based ecological momentary assessment study of physical activity and sedentary behavior. Innov Aging.

[32] Ramsey AT, Wetherell JL, Depp C, Dixon D, Lenze E (2016). Feasibility and acceptability of smartphone assessment in older adults with cognitive and emotional difficulties. J Technol Hum Serv.

[33] Mindlis I, Rodebaugh TL, Kiosses D, Reid MC (2026). Feasibility and acceptability of ecological momentary assessments in depressed older adults with multimorbidity. J Appl Gerontol.

[34] Birati Y, Tzemah-Shahar R (2026). Barriers to digital health adoption in older adults: scoping review informed by innovation resistance theory. J Med Internet Res.

[35] Mao A, Tam L, Xu A, Osborn K, Sheffrin M, Gould C, Schillinger E, Martin M, Mesias M (2022). Barriers to telemedicine video visits for older adults in independent living facilities: mixed methods cross-sectional needs assessment. JMIR Aging.

