# Supplementary material for: Smartphone-Based Ecological Momentary Assessment Among Community-Dwelling Older Adults: Observational Feasibility and Acceptability Study
Source: JMIR Form Res. 2026 Jul 8;10:e94949. doi: 10.2196/94949 (PMC13392534; doi:10.2196/94949)
Supplement: Multimedia Appendix 1 [file formative_v10i1e94949_app1.docx]

Multimedia Appendix 1. Abbreviated Mental Test (AMT)- Singapore Version

| Item | Question |
| --- | --- |
| 1 | What is the year? |
| 2 | What is the time? (within 1 hour) |
| 3 | What is your age? |
| 4 | What is your date of birth? |
| 5 | What is your home address? |
| 6 | Where are we now? |
| 7 | Who is our country’s Prime Minister? |
| 8 | Memory phase “37 Bukit Timah Road” |
| 9 | Count backwards from 20 to 1 |
| 10 | Recall memory phrase |

Reference: Ministry of Health S. Clinical Practice Guidelines: Dementia. Ministry of Health, Singapore, 2013.
