# Supplementary material for: Smartphone-Based Ecological Momentary Assessment Among Community-Dwelling Older Adults: Observational Feasibility and Acceptability Study
Source: JMIR Form Res. 2026 Jul 8;10:e94949. doi: 10.2196/94949 (PMC13392534; doi:10.2196/94949)
Supplement: Multimedia Appendix 2 [file formative_v10i1e94949_app2.docx]

Multimedia Appendix 2. Baseline Assessment Questionnaire

| Section | Item | Question/ Response Options |
| --- | --- | --- |
| 1. Personal demographic | Participant ID | Free text |
|  | Sex | Male or Female |
|  | Race | Chinese, Malay or Indian |
|  | Age | Free text |
| 1. Medical History | Diagnosis history | Have you ever been diagnosed with or currently have stroke, heart attack, kidney failure, or cancer? |
| 1. Education level | Highest education attained | No formal qualification/Lower primary, Primary (PSLE), Secondary (‘O’/ ‘N’ level), ITE/NTC, A-level/polytechnic/diploma, University, or Prefer not to answer |
| 1. Mental Health | 1. Tiredness | Not at all, A little, Quite a bit, Very, or Extremely |
|  | 1. Happiness | Not at all, A little, Quite a bit, Very, or Extremely |
|  | 1. Worried | Not at all, A little, Quite a bit, Very, or Extremely |
|  | 1. Stressed | Not at all, A little, Quite a bit, Very, or Extremely |
|  | 1. Lonely/Isolated | Not at all, A little, Quite a bit, Very, or Extremely |
|  | 1. Boredom | Not at all, A little, Quite a bit, Very, or Extremely |
|  | 1. Overall mental wellbeing | On a scale from 1 to 5, how would you rate your current mental wellbeing?  1 (poor)-2-3-4-5 (excellent) |
