# Supplementary material for: Smartphone-Based Ecological Momentary Assessment Among Community-Dwelling Older Adults: Observational Feasibility and Acceptability Study
Source: JMIR Form Res. 2026 Jul 8;10:e94949. doi: 10.2196/94949 (PMC13392534; doi:10.2196/94949)
Supplement: Multimedia Appendix 3 [file formative_v10i1e94949_app3.docx]

Multimedia Appendix 3. Ecological Momentary Assessment (EMA) Schedule and Items

| Prompts | Measurements | Time | EMA questions | Response options |
| --- | --- | --- | --- | --- |
| 1 | Sleep | 7am | 1. How would you rate your overall sleep quality? | 1-5 Likert scale  (1=poor, 5=excellent) |
|  |  |  | 2. How many hours did you sleep last night? | Less than 5 hours; Between 5 and 6 hours; Between 6 and 7 hours; Between 7 and 8 hours;  More than 8 hours |
| 2,3,4 | Emotional states | 3 times:  9am, 12pm, 4pm | 1. How tired do you feel right now | Not at all;  A little;  Quite a bit;  Very;  Extremely |
|  |  |  | 2. How happy do you feel right now |  |
|  |  |  | 3. How worried do you feel right now |  |
|  |  |  | 4. How stressed do you feel right now |  |
|  |  |  | 5. How lonely/isolated do you feel right now |  |
|  |  |  | 6. How bored do you feel right now |  |
|  |  |  | 7. How would you rate your overall mental wellbeing at this moment. | 1-5 Likert scale  (1=poor,  5=excellent) |
|  | Perceived Neighbourhood Environment | 3 times:  9am, 12pm, 4pm | 1. How safe do you feel in your current location today? | 1=5 Likert scale  (1=Very unsafe; 5=Very safe) |
|  |  |  | 2 Is there a green space or park nearby where you are right now? | Yes/no |
|  |  |  | 3. Are you experiencing any loud noises or disruptions in your neighbourhood today? | Yes/no |
|  |  |  | 4. Do you feel socially connected to your neighbours and community members at this moment | Yes/no |
|  |  |  | 5. Is it convenient to walk or exercise in your neighbourhood right now? | Yes/No |
|  |  |  | 6. How would you rate your overall neighbourhood right now? | 1-5 Likert scale  (1=poor; 5=excellent) |
| 5 | Physical activity | 7pm | 1. Have you engaged in any physical activity today? | Yes/No |
|  |  |  | 2. How many minutes of physical activity have you done today? | 0 minutes;  15 minutes or less;  16-30 minutes;  31-60 minutes; More than 60 minutes |
|  | Community cohesion | 7pm | 1. Did you interact with neighbours or other community members today? | Yes/No |
|  | Screen use | 7pm | 1. Did you look at a screen while eating today? | Yes/No |
|  |  | 7pm | 2. How much time have you spent on screens (e.g., TV, computer, smartphone, tablet) today? | 0 hours; Less than 1 hour; 1-2 hours;2-3 hours;  More than 3 hours |
|  | Daytime Napping | 7pm | 1. How many hours did you nap today? | 0 hours; Less than 1 hour;1-2 hours;2-3 hours; More than 3 hours |
