# Supplementary figures and images for: Smartphone-Based Ecological Momentary Assessment Among Community-Dwelling Older Adults: Observational Feasibility and Acceptability Study
Source: JMIR Form Res. 2026 Jul 8;10:e94949. doi: 10.2196/94949 (PMC13392534; doi:10.2196/94949)

Multimedia Appendix 4. Daily EMA Prompts (1-5) Administered via RealLife Exp App


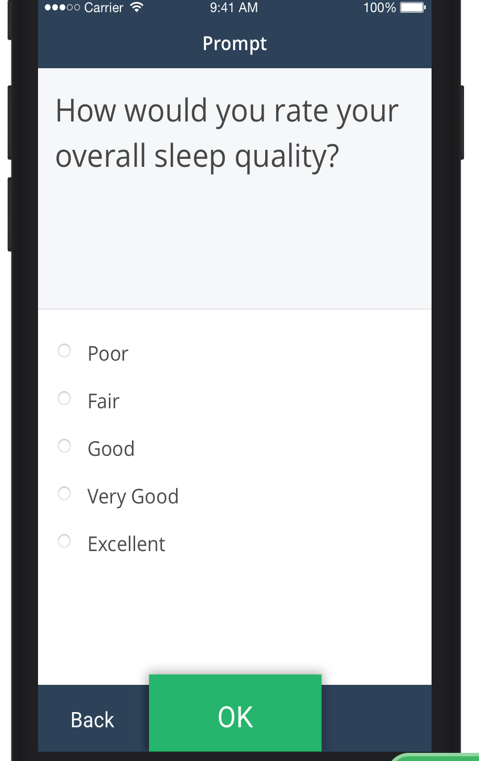

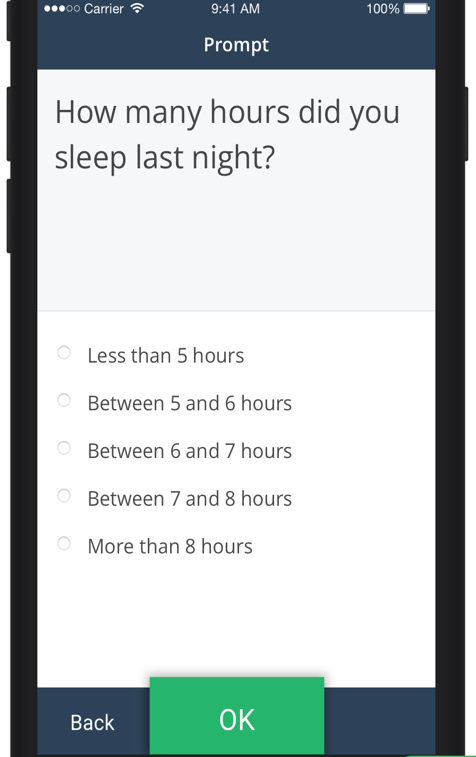

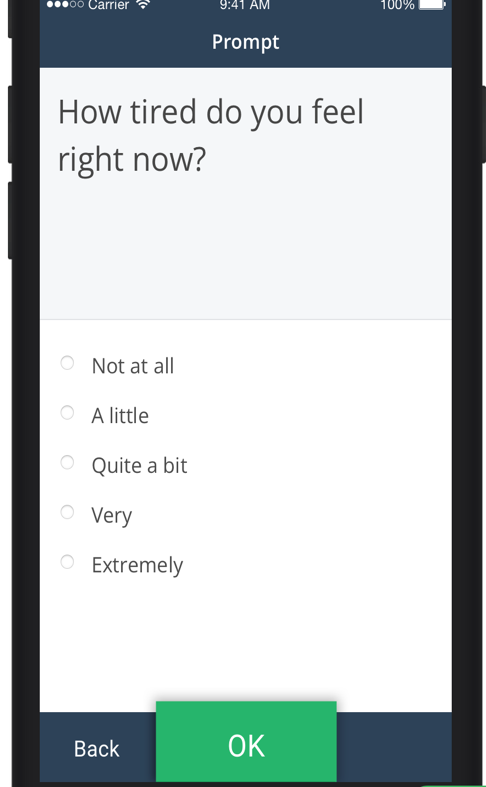


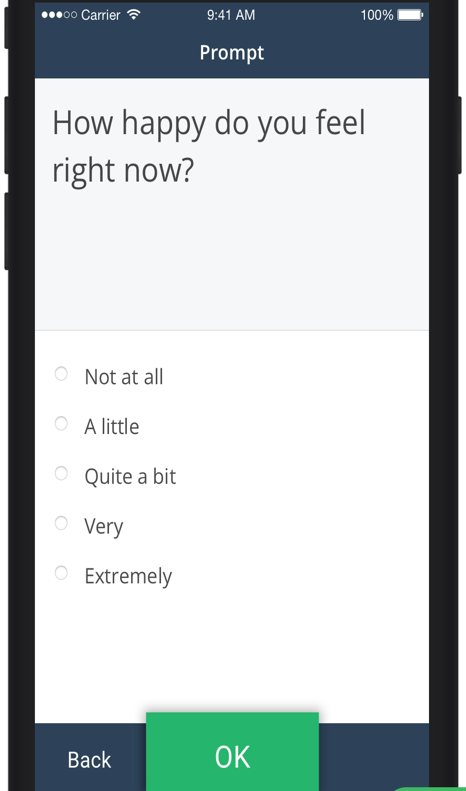

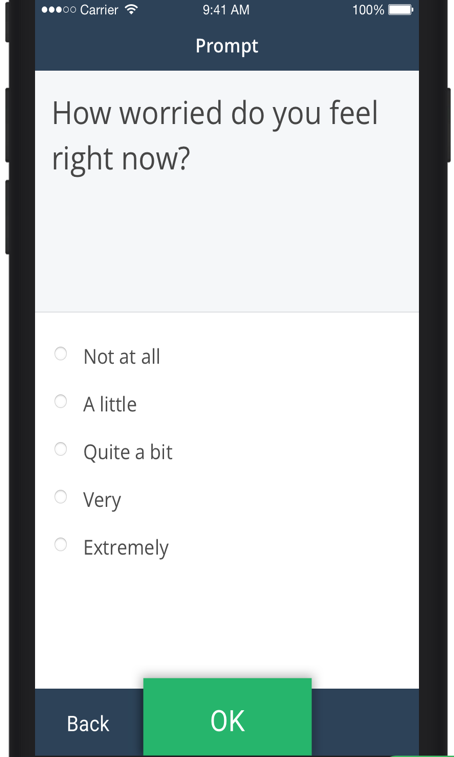

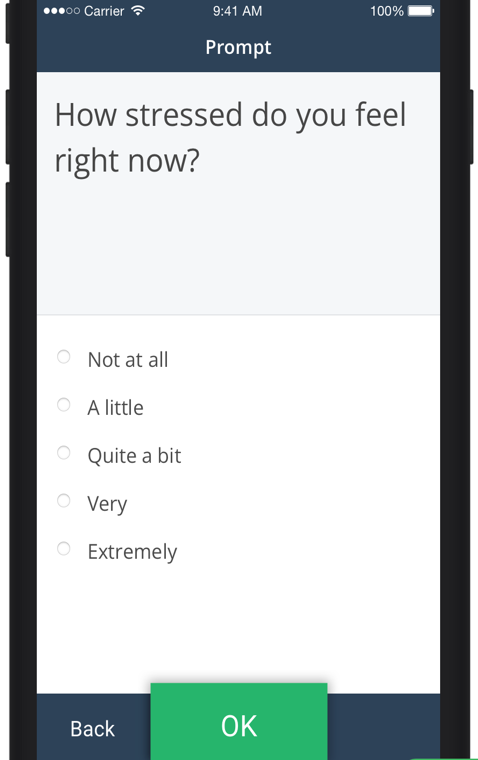


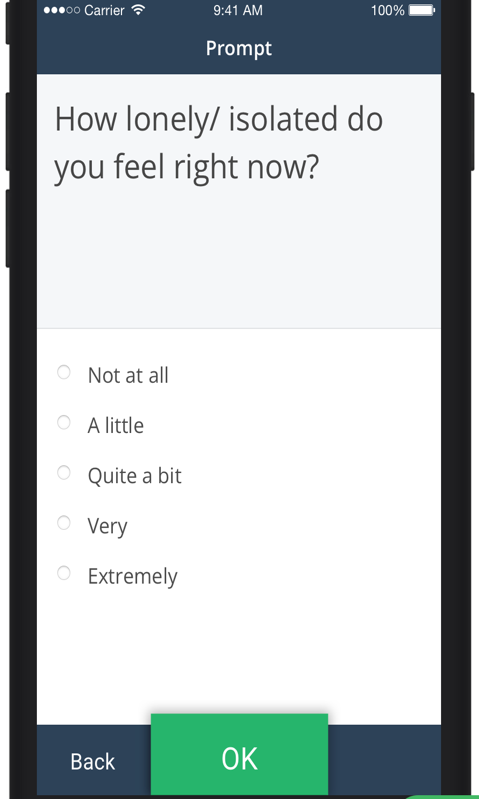

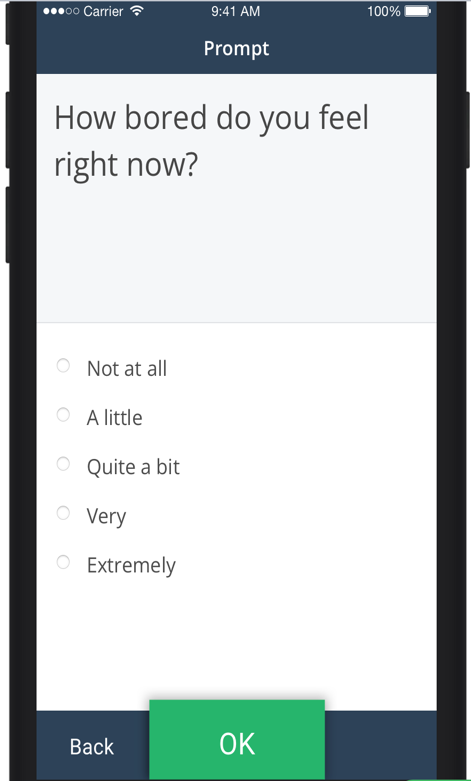

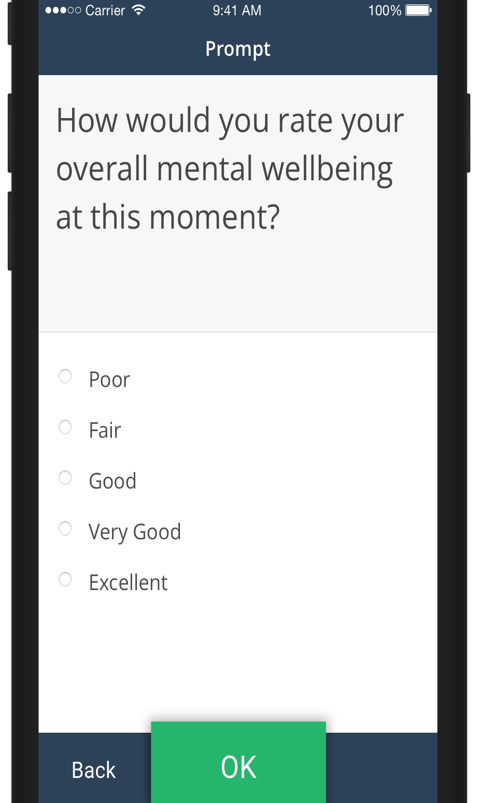


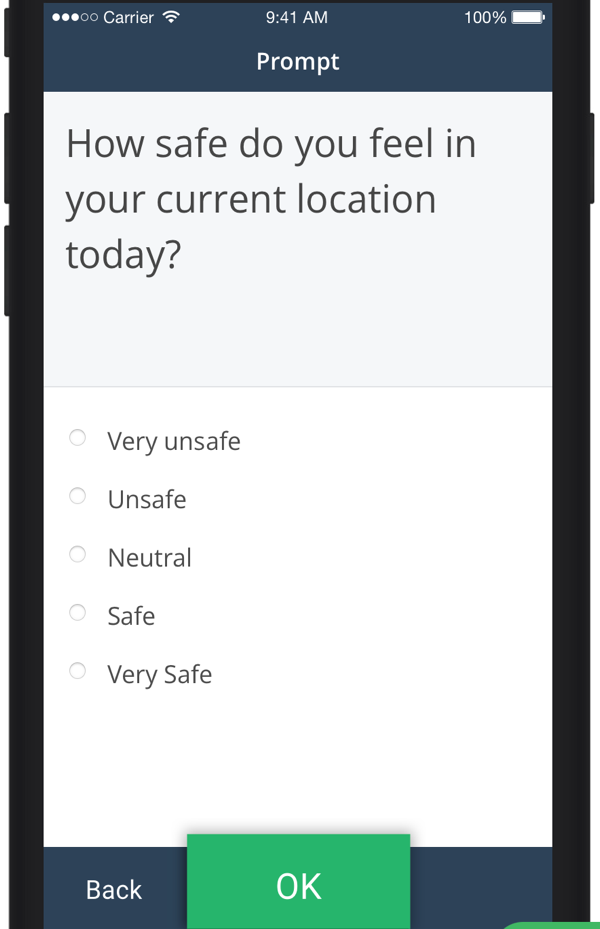

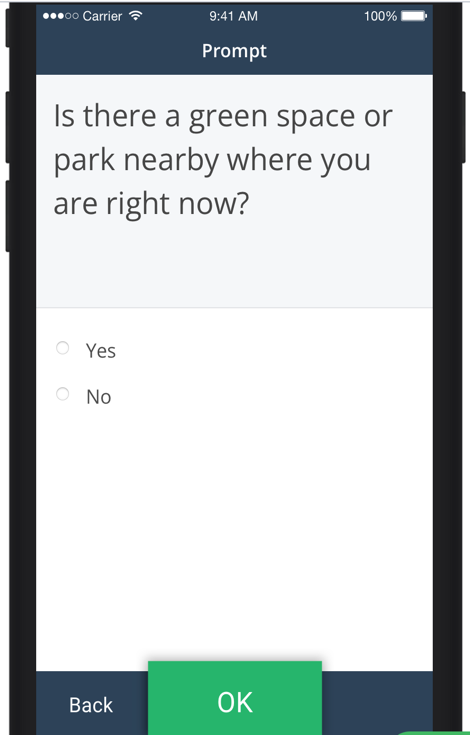

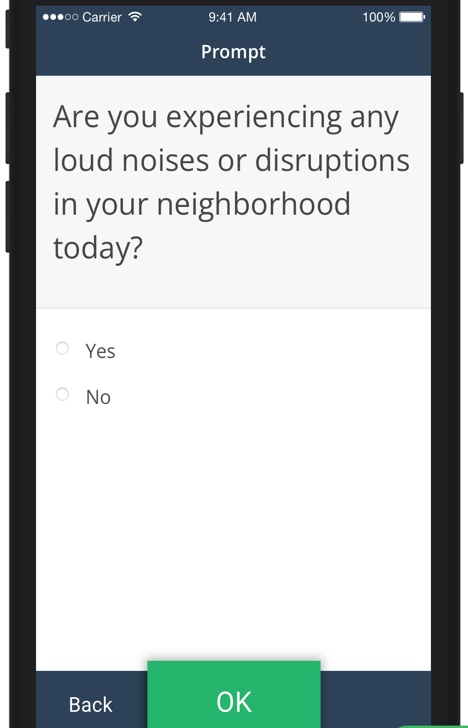


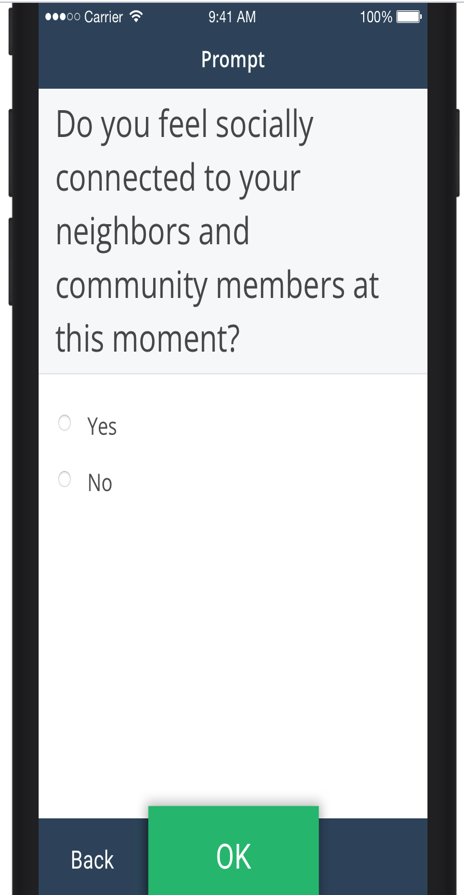

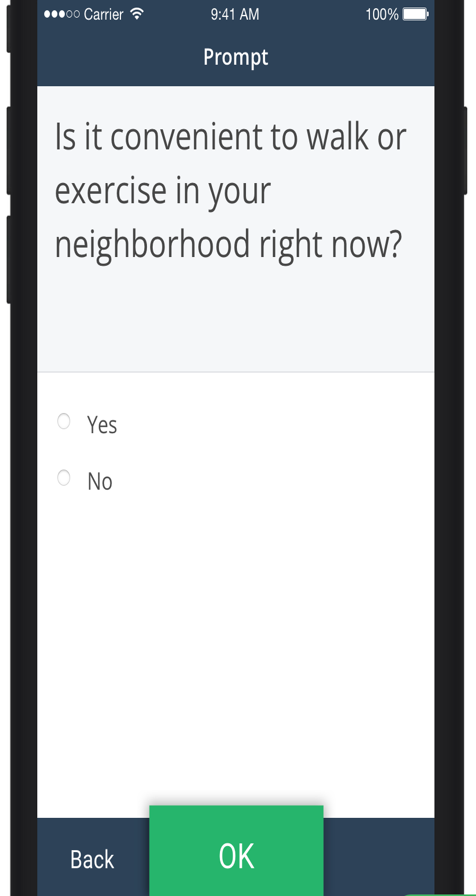

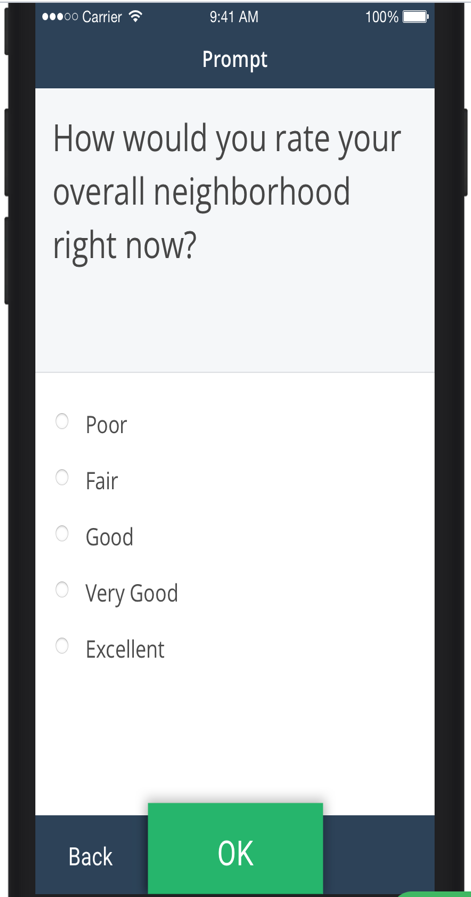


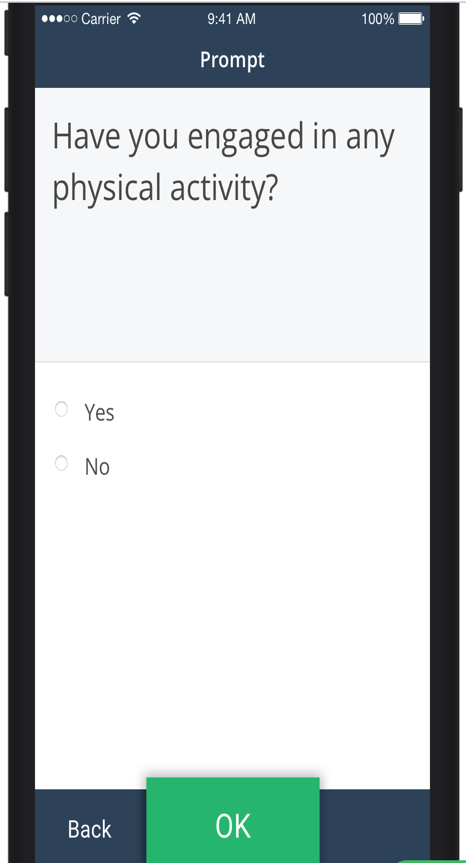

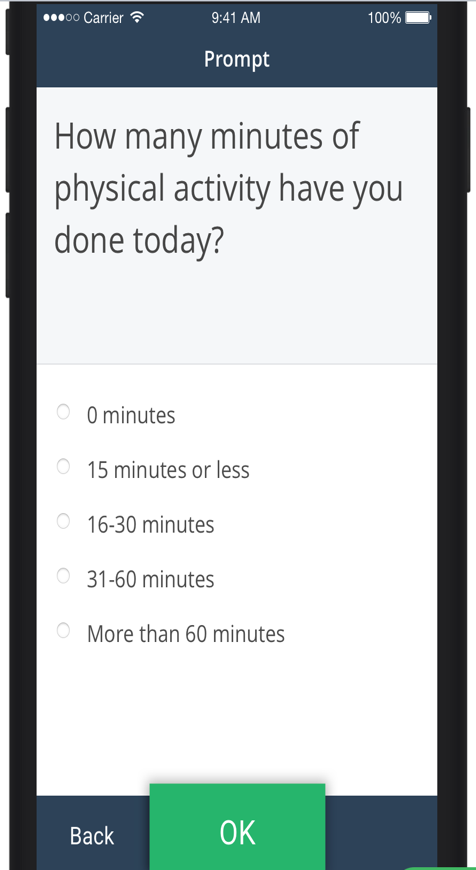

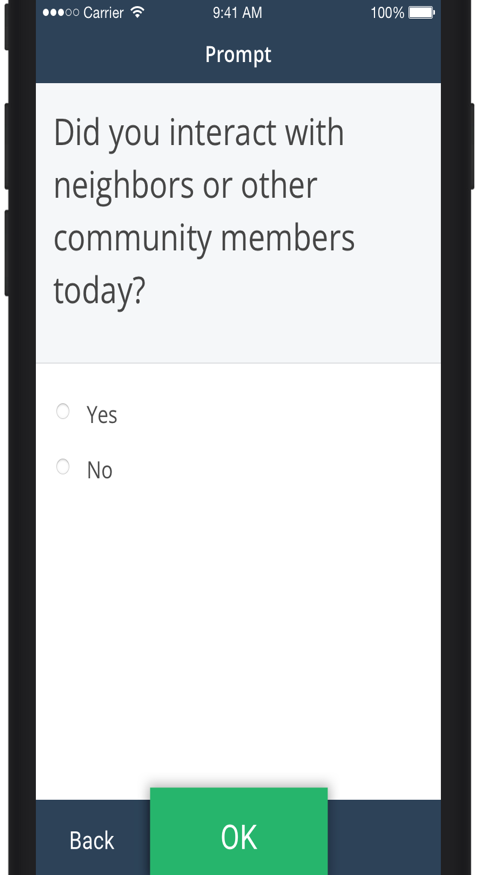

Supplement: Multimedia Appendix 4 [file formative_v10i1e94949_app4.docx]
