# Supplementary material for: Smartphone-Based Ecological Momentary Assessment Among Community-Dwelling Older Adults: Observational Feasibility and Acceptability Study
Source: JMIR Form Res. 2026 Jul 8;10:e94949. doi: 10.2196/94949 (PMC13392534; doi:10.2196/94949)
Supplement: Multimedia Appendix 6 [file formative_v10i1e94949_app6.docx]

Multimedia Appendix 6. EMA response timing and response quality metrics among completed EMA prompts.

| Domain | Metrics | Result |
| --- | --- | --- |
| Response timing | Median response latency, minutes (IQR) | 11.1 minutes (IQR 1.0-33.7) |
|  | Completed within 15 minutes of prompt | 4,968/9,007 (55.2%) |
|  | Completed within 1 hour of prompt | 7,696/9,007 (85.4%) |
|  | Completed within 5 hours (full window) | 9,007/9,007 (100%) |
| Prompt completion time^a^ | Median prompt completion time, seconds (IQR) | 47 seconds (32-67) |
|  | Extremely short prompts (<10 seconds) | 253/8,992 (2.8%) |
| Potential repetitive responding^b^ | Prompts with straight-lining | 61/5,460 (1.1%) |
|  | Participants with any straight-lining | 22/130 (16.9%)^c^ |

^a^Prompt completion time data were unavailable for 15/9,007 (0.2%) completed prompts due to app recording errors.

^b^Straight-lining was assessed only across same-scale emotional state items (tiredness, happiness, worry, stress, loneliness, boredom) in daytime EMA prompts (prompts 2, 3, and 4). Sleep and lifestyle prompts were excluded due to heterogeneous response formats.

^c^16.9% reflects participants with at least one instance of straight-lining across 42 prompts; the overall rate at the prompt level was 1.1%.
